# Earnings and work loss after colon and rectal cancer: a Swedish nationwide matched cohort study

**DOI:** 10.1016/j.eclinm.2024.102770

**Published:** 2024-08-06

**Authors:** S.E. Boman, I. Hed Myrberg, G. Bruze, A. Martling, C. Nordenvall, P.J. Nilsson

**Affiliations:** a Clinical Epidemiology Division, Department of Medicine Solna, Karolinska Institutet, Stockholm, Sweden; b Department of Medical Epidemiology and Biostatistics, Karolinska Institutet, Stockholm, Sweden; c Department of Molecular Medicine and Surgery, Karolinska Institutet, Stockholm, Sweden; d Department of Colorectal Surgery, Karolinska University Hospital, Stockholm, Sweden

**Keywords:** Colorectal cancer, Financial toxicity, Work loss, Epidemiology, Earnings

## Abstract

**Background:**

Colorectal cancer is common and prognosis is improving. The conditions of survivors of treatment, including financial consequences, are thus important. The aim of this study was to quantify loss of earnings and work loss in working-age patients with colon and rectal cancer relative to matched comparators.

**Methods:**

The study utilised data from the CRCBaSe database that is generated from the nationwide Swedish ColoRectal Cancer Register and includes data from several Swedish nationwide registers. The study period was 1995–2020 for rectal cancer patients and 2007–2020 for colon cancer patients. A retrospective population-based nationwide cohort study on earnings, disposable income, and work loss, in survivors of stage I-III colorectal cancer treatment was undertaken. Median regression was used to analyse earnings and disposable income, and logistic regression to analyse the probability of work loss.

**Findings:**

A cohort of 8863 colorectal cancer survivors diagnosed before 2017 and 52,514 comparators matched on birth year, legal sex, and county of residence, was analysed. There was a clear reduction in earnings between the calendar year prior to and the calendar year after diagnosis, from € 31,319 to € 23,924 for colon cancer patients and from € 32,636 to € 22,647 for rectal cancer patients, and earnings never fully recovered during the 5-year follow-up. Disposable income was practically unaltered. The probability of work loss increased in the calendar year of diagnosis, from 29.8% to 25.3% the previous year to 83.3% and 84.4% for colon and rectal cancer patients respectively, and never fully recovered. The probability of work loss was similar between colon and rectal cancer survivors, but was higher among patients with rectal cancer who had received neoadjuvant therapy.

**Interpretation:**

This study shows that despite an extensive welfare system providing maintained disposable income, there is a financial burden in the form of increased risk of work loss and a reduction in earnings among survivors of colorectal cancer.

**Funding:**

The study was supported by the 10.13039/501100002794Swedish Cancer Society, the Swedish Cancer and Allergy Foundation, and the Stockholm Cancer Society, and supported by grants provided by the Regional Agreement on Medical Training and Clinical Research (ALF) between the Stockholm County Council and 10.13039/501100004047Karolinska Institutet.

**Research in context**

**Evidence before this study**

During March 2022, a PubMed search, without language restrictions, using terms related to colorectal cancer, financial toxicity, financial hardship, earnings, and work loss was performed. It indicated that the current knowledge of financial toxicity in colorectal cancer survivors was limited or non-existent. Advances in treatment have led to improved prognosis for many cancer patients including patients diagnosed with colorectal cancer. Consequently, quality-of-life and other aspects of survivorship have become increasingly important. The financial implications of a cancer diagnosis have therefore gained increased interest in the literature over the recent years, but most studies have emerged from North America and only few concerns colorectal cancer. In addition, available evidence on a nationwide population level was limited.

Colorectal cancer is often considered a single entity although several differences exist concerning molecular signatures, treatment and prognosis between colon and rectal cancer. Mostly, previous studies on financial implications of colorectal cancer have not subdivided patients into colon and rectal cancer.

When this study was conceived, CRCBaSe, which consists of a linkage between the Swedish Colorectal Cancer Register (SCRCR) and several Swedish population-based registers, had recently been finalized. Thus, an opportunity arose to investigate financial implications such as earnings, disposable income and work loss on a population level.

**Added value of this study**

This study provides nationwide data from high-quality registers on the effects of stage I-III colon and rectal cancer on earnings, disposable income and work loss over a five-year period following diagnosis. Including matched comparators from the general population enables a comparison not only between colon and rectal cancer patients but also with the background population. The study is set in a European country with a highly developed welfare system and low out-of-pocket medical costs. This study adds significantly to the body of evidence describing financial toxicity from colon and rectal cancer. Ongoing initiatives from, e.g., the European Society of Medical Oncology (ESMO) on how to manage financial toxicity may be aided by the nationwide population-based data provided. Furthermore, this study reveals potential differences between colon and rectal cancer patients also regarding the financial impact of disease, implicating that future research should separate these entities.

**Implications of all the available evidence**

The incidence of early-onset colorectal cancer (<50 years at diagnosis) is increasing, which will have implications for the society. This nationwide study of colorectal cancer patients of working age makes it possible to estimate work loss and its consequences. Earnings and work loss reflect financial impact on both a societal and individual level, whereas disposable income can be viewed as a direct measure of financial implications from disease on a personal level. Additionally, subjective financial distress is an important factor. Irrespective of healthcare and welfare system within a country, work loss and reduced earnings can lead to undesirable outcomes both for the affected individual and the society. Efforts to promote the return to work in colorectal cancer survivors are encouraged.

## Introduction

Colorectal cancer (CRC) is common, with incidence rates for colon and rectal cancer in the European Union around 50 and 25 per 100,000 persons, respectively.^1^^,^^2^ Early-onset CRC, i.e., diagnosis in individuals younger than 50 years, is increasing in certain high-income countries,^3^ and survival among individuals diagnosed with CRC is improving globally.^2^ Hence, survivors of CRC may constitute an increasing proportion of working age citizens.

The economic consequences of a cancer diagnosis, both on an individual and a societal level, can be substantial. The term financial toxicity is used to refer both to objective financial burden and subjective financial distress associated with cancer diagnosis and treatment.^4^ Cost of cancer care can vary considerably depending on the country and its healthcare system, but it is recognized that a cancer diagnosis can also impair work ability.^5^ Further, an association between financial toxicity and psychological well-being in cancer survivors has been reported.^6^ Financial toxicity has been previously studied in various types of cancer.^7^, ^8^, ^9^ Smaller studies from Ireland, Germany, the U.S, and China, all indicate that CRC is associated with financial toxicity.^10^, ^11^, ^12^, ^13^ In Sweden, out-of-pocket costs for patients are limited because of a government-funded high-cost protection^14^ placed individually on care-provider visits, treatment (including medications), and travel, and in this paper the focus will be on loss of income and work loss.

Cancer of the colon and cancer of the rectum differ regarding molecular signatures and treatment strategies. Rectal cancer patients are more commonly treated with neoadjuvant (chemo)radiotherapy, and patients with rectal cancer more commonly receive a permanent ostomy. These factors may impact a patient's quality of life and the ability of the patient to return to work after the cancer has been cured.

The aim of this study was to quantify earnings, disposable income, and work loss (the sum of sick leave and disability pension) in working-age patients with colon and rectal cancer relative to matched comparators. We also aimed to contrast colon and rectal cancer patients (vis-a-vis their respective comparators), with the hypothesis that rectal cancer patients experience greater loss of income and/or work than colon cancer patients.

## Methods

### The colorectal cancer database (CRCBaSe)

This study utilized data from the Colorectal Cancer database (CRCBaSe), a Swedish national research database linking information from several national registers for patients diagnosed with CRC and matched comparators from the general population.^15^ CRCBaSe is generated from the Swedish Colorectal Cancer Register (SCRCR), which contains nearly all rectal and colon cancer patients in Sweden diagnosed from 1995 to 2007, respectively. Misclassification bias of exposure and outcome was minimized by using data sources with high validity. SCRCR contains detailed information on tumor and treatment characteristics, and has been shown to have a completeness of above 98%.^16^ CRCBaSe also includes information from several registers at the National Board of Welfare and Statistics Sweden, including the Longitudinal Integrated Database for Health Insurance and Labour Market Studies (LISA), from which information regarding education, earnings, disposable income, and work loss was retrieved.^17^

The study was approved by the Regional Board of the Ethical Committee in Stockholm, Sweden (DNR: 2014/71-31, 2018/328-32, 2021-00342, 2023-03305-02).

### Study population

The study population aimed to target CRC patients and matched comparators of the working population, and inclusion criteria were curatively treated stage I-III CRC (excluding appendiceal cancer) in patients who were between the ages of 20 up to and including 60 at the date of diagnosis. To ensure adequate follow-up time for all included patients, only patients who were diagnosed before 2017 were included. CRCBaSe contains six comparators per patient, who are matched with respect to a patient's birth year, legal sex, and county of residence, and who are alive at the time of diagnosis. Additionally, comparators cannot have a previous or current CRC at the matching date (the patient's date of diagnosis). No exclusions were made based on year of diagnosis, meaning that rectal cancer patients were included from 1995 and onwards whereas colon cancer patients were included from 2007 and onwards. This resulted in a cohort of 3591 colon cancer and 5272 rectal cancer patients with 21,266 and 31,248 comparators, respectively.

### Variables

Data on taxable earnings, disposable income, sick leave, and disability pension for patients and comparators were retrieved from the LISA database. The earnings data originate from reports made by employers to the Swedish Tax Agency listing the total taxable earned gross income of each employee in a calendar year. This measure of earnings captures changes in income due to, for example, a job change within or between companies, shifts from permanent to temporary positions, or shifts from full-time to part-time positions.

Disposable income is defined as the total income, including capital income, returns on assets, and government transfers such as child benefits and disability pension, that a person receives minus paid income taxes. Both annual taxable earnings and disposable income were included in this study as outcomes, since earnings are more closely related to work ability and disposable income is primarily a measure of an individual's living standards.

The Swedish welfare system provides compensation for sick leave and disability pension, which may be complete or partial. Sick leave is reimbursed by the employer from day 2 to day 14. Episodes longer than 14 days are recorded by the Social Insurance Agency, which reimburses the employee due to lost income from day 15 and onwards. An employee with a 25% reduced work ability or more (as evaluated by a physician) that is expected to last for at least 1 year, may receive disability pension (on a time-limited or permanent basis).

Our measure of work loss is the sum of sick leave and disability pension. Combining these two types of benefits gives a unified measure of work loss that is comparable over time and insensitive to institutional changes that may move individuals between these two benefit systems. The presence of work loss in a calendar year was coded as a binary variable with the value of 1 if an individual received benefits for sick leave or disability pension in that year.

Data on earnings, disposable income, and work loss were collected for each individual from the calendar year prior to CRC diagnosis/date of matching until five years after diagnosis. Data on the highest obtained education was categorized into three groups (≤9 years, 10–12 years, and >12 years) corresponding to primary school, upper secondary school, and university/college. Earnings and disposable income were adjusted for inflation and converted to euros (€), using the year 2020 as a reference year.

### Statistical methods

Annual earnings and disposable income were compared between CRC patients and their matched comparators using median regression with robust and clustered standard errors, taking into account the longitudinal follow-up, implemented in Stata Version 18 using the qreg2 function.^18^ It was required that each individual had financial data available for any of the years 0–5, with year 0 denoting the year of diagnosis. A sensitivity analysis restricted to individuals alive at least 3 years after diagnosis was performed. Patient data was censored in case of cancer recurrence, emigration, or reaching end-of-study (1st January 2021), and comparator data was censored in case of colorectal cancer diagnosis or reaching end-of-study.

The annual probability of work loss was compared between CRC patients and comparators by applying logistic regression with robust and clustered standard errors, implemented in R using glm^19^ and vcovCL from the ‘sandwich’ package.^20^^,^^21^ Similarly, sick leave and disability pension were analyzed as separate outcomes in a supplementary analysis.

In all regression models, the predictor was a three-way interaction term between cancer exposure, cancer location (colon or rectum), and years since the year of diagnosis/matching. Confounding bias was addressed by matching on age (legal) sex, and geographic region, and by adjusting for relevant covariates in all analyses. Adjustments were made for sex, calendar date of the cancer diagnosis (using restricted cubic splines with two internal knots placed at distribution quantiles), the highest obtained education level, and age at the time of diagnosis/matching. Analyses were done as a complete case analysis, by excluding individuals with missing data during formation of the study population. The reference levels were chosen to be mean levels, with sex being coded as −1 and 1 with a reference level of 0. The middle education level was chosen as reference level, and the middle of 2020 was chosen as a reference level for the calendar date of diagnosis/matching in order to match the reference years for inflation and currency conversion. The estimated earnings, disposable income, and probabilities of work loss thus represent outcomes with covariates at these reference levels. The difference in disposable income, earnings, and probability of work loss between patients and comparators was contrasted for colon vs rectal cancer by applying Wald tests for three-way interaction terms between cancer exposure, cancer location, and year since the year of diagnosis/matching.

For each outcome, a three-way interaction between cancer exposure, sex, and years from diagnosis/matching was added, but omitted from the final models due to lack of statistical significance.

A significance level of 5% was used for all statistical hypothesis testing.

### Ethics

Ethical approval was given by the Regional Board of the Ethical Committee in Stockholm, Sweden (DNR: 2014/71-31, 2018/328-32, 2021-00342) and the study was conducted in accordance with the ethical standards described. The need to obtain informed consent was waivered in our ethical permit for reasons related to feasibility.

### Role of funding source

No funding sources had a role in any part of this study including data acquisition, study design, analysis or writing of the manuscript.

## Results

**Fig. 1:**
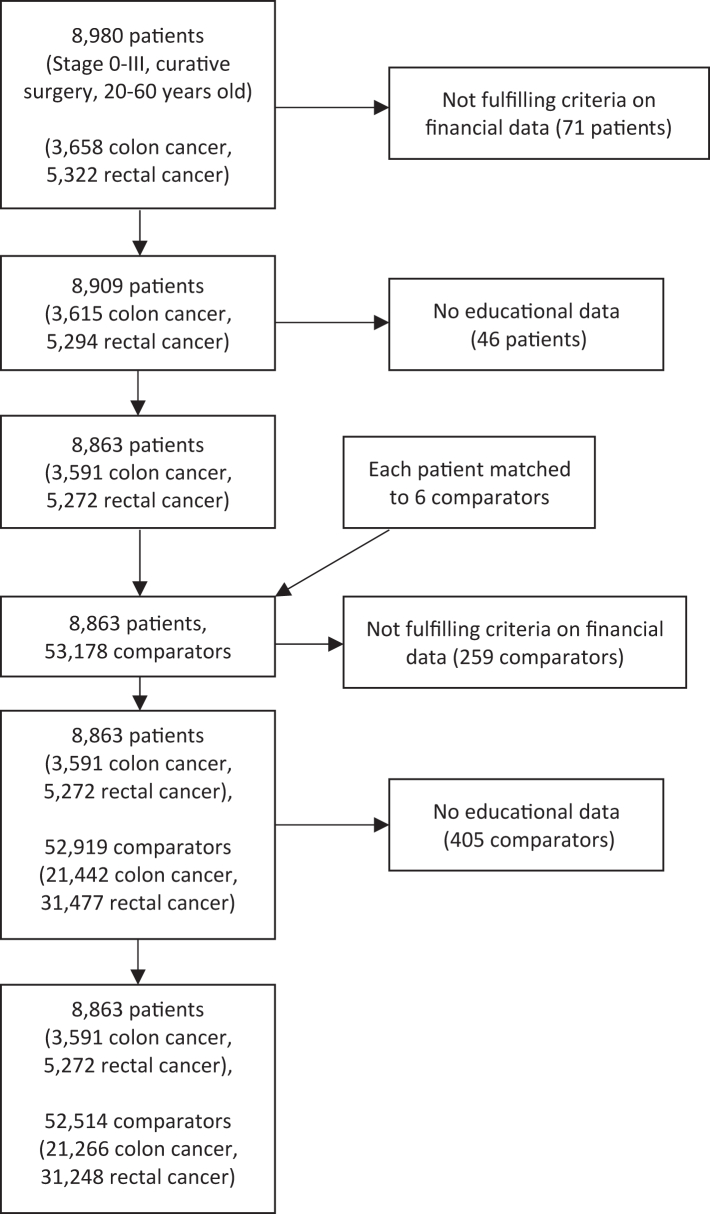
An exclusion flow chart for the study population.

In total, 3591 colon cancer and 5272 rectal cancer patients with 21,266 and 31,248 comparators, respectively, were identified (Fig. 1). In Table 1, demographic and treatment characteristics are presented. There was no substantial difference in age at diagnosis between colon and rectal cancer patients, whereas male sex was more common in rectal cancer. A higher education level was more common in colon cancer patients compared with rectal cancer patients (university level 34.7% vs 30.9%, respectively).

**Table 1:** Characteristics of 8863 Swedish colorectal cancer patients and 52,514 comparators matched by age, sex and county of residence.

Variable	Colon cancer		Rectal cancer	
	Patient (n = 3591)	Comparator (n = 21,266)	Patient (n = 5272)	Comparator (n = 31,248)
**Follow-up years**^a^				
Mean (SD)	4.35 (1.24)	4.63 (0.80)	4.33 (1.32)	4.74 (077)
**Age at diagnosis/matching**				
Mean (SD)	52.1 (7.55)	52.1 (7.52)	52.8 (6.73)	52.8 (6.73)
Median [Q1, Q3]	54.0 [48.0, 58.0]	54.0 [48.0, 58.0]	55.0 [50.0, 58.0]	55.0 [50.0, 58.0]
**Sex**				
Male	1844 (51.4%)	10,896 (51.2%)	2986 (56.6%)	17,658 (56.5%)
Female	1747 (48.6%)	10,370 (48.8%)	2286 (43.4%)	13,580 (43.5%)
**Stage**^b^				
Stage 0	7 (0.2%)	–	111 (2.9%)	–
Stage 1	601 (17.3%)	–	1136 (29.7%)	–
Stage 2	1307 (37.6%)	–	1068 (27.9%)	–
Stage 3	1559 (44.9%)	–	1512 (39.5%)	–
Missing	117	–	1445	–
**ASA class**				
ASA class 1	1304 (38.2%)	–	1158 (42.2%)	–
ASA class 2	1742 (51.0%)	–	1360 (49.5%)	–
ASA class 3	353 (10.3%)	–	221 (8.0%)	–
ASA class 4	16 (0.5%)	–	7 (0.3%)	–
Missing	176	–	2526	
**Education**				
Primary school	578 (16.1%)	3631 (17.1%)	1206 (22.9%)	7068 (22.6%)
Upper secondary school	1766 (49.2%)	10,153 (47.7%)	2437 (46.2%)	14,453 (46.3%)
University	1247 (34.7%)	7482 (35.2%)	1629 (30.9%)	9727 (31.1%)
**Calendar year of patient diagnosis**				
1995–2006	12 (0.3%)	71 (0.3%)	2477 (47.0%)	14,679 (47.0%)
2007–2010	1198 (33.4%)	7102 (33.4%)	935 (17.7%)	5545 (17.7%)
2011–2013	971 (27.0%)	5748 (27.0%)	818 (15.5%)	4857 (15.5%)
2014–2017	1410 (39.3%)	8345 (39.2%)	1042 (19.8%)	6167 (19.7%)
**Neoadjuvant treatment**				
No neoadjuvant treatment	3494 (97.3%)	–	1410 (26.7%)	–
Neoadjuvant radiotherapy only	8 (0.2%)	–	2934 (55.7%)	–
Neoadjuvant chemotherapy only	72 (2.0%)	–	24 (0.5%)	–
Neoadjuvant chemoradiotherapy	17 (0.5%)	–	904 (17.1%)	–
**Adjuvant treatment**^c^				
No adjuvant treatment	1949 (54.3%)	–	4366 (82.8%)	–
Adjuvant chemotherapy only	1642 (45.7%)	–	904 (17.1%)	–
**Surgery type**				
Right-sided hemicolectomy	1304 (37.5%)	–	1 (0.0%)	–
Left-sided hemicolectomy	530 (15.3%)	–	0 (0%)	–
Sigmoideum resection	997 (28.7%)	–	3 (0.1%)	–
Total colectomy	269 (7.7%)	–	6 (0.1%)	–
Hartmann's procedure	48 (1.4%)	–	131 (2.5%)	–
Abdominoperineal resection	3 (0.1%)	–	1626 (31.2%)	–
Anterior resection	265 (7.6%)	–	3294 (63.2%)	–
Other	59 (1.7%)	–	154 (3.0%)	–
Missing	116	–	57	–

Percentages are calculated among non-missing.

a Follow-up is recorded as the amount of whole years passed between records in the LISA database, between the year of diagnosis and the final recorded year before censorship or completion of follow-up (5 years).

b If staging by a pathologist has been done, this is the pathological staging, otherwise it's the clinical staging. Stage 3 is defined as a tumor that has spread into at least one lymph node, but which hasn't metastasized. Stages 0–2 are defined as a stage T0, T1-T2, and T3-T4 tumor respectively, with no lymph node metastasis or metastasis.

c Two rectal cancer patients received adjuvant radiotherapy and are not included.

Because SCRCR contains rectal cancer patients from 1995 and colon cancer patients from 2007, the rectal cancer patients were on average diagnosed at an earlier date. Advanced postoperative stage was more common in colon cancer patients compared to rectal cancer patients (Stage III 45% vs 40% of non-missing), while use of neoadjuvant therapy was less common for patients of colon cancer (2.7% vs 73.3%). Adjuvant chemotherapy was more commonly used in colon cancer patients (46% vs 17%).

**Fig. 2:**
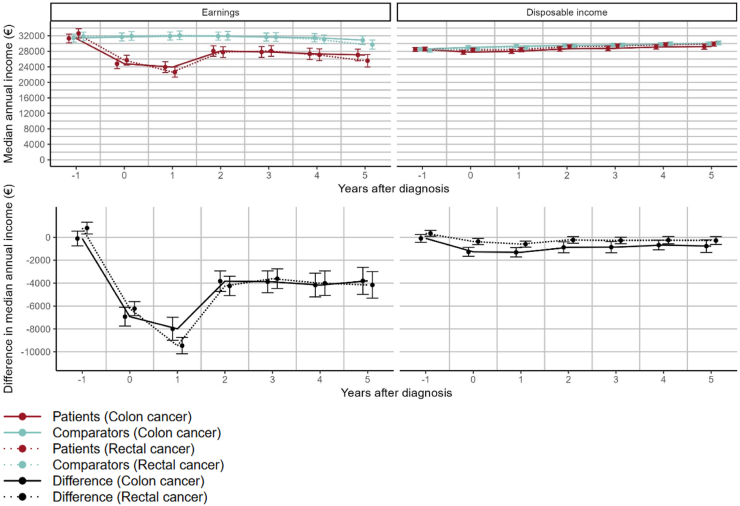
Median annual income (top panel), and difference in median annual income between CRC cases and matched controls (bottom panel), starting the calendar year prior to diagnosis and ending in the fifth year after diagnosis, with year 0 being the calendar year of diagnosis, converted to euros and inflation-adjusted to represent the value of a euro in the year 2020. The point estimates (but not the confidence intervals) in the graphs of the bottom panels are the differences between the respective patient and comparator groups from the top panels. The levels and differences are adjusted for age, sex, calendar date, and education level, with reference levels being age 52.5 years, calendar date 1st of July 2020, and upper secondary school education. Vertical lines show 95% confidence intervals.

The median annual earnings and disposable income, and differences between patients and comparators, are shown in Fig. 2. Median earnings drop significantly in the years following both colon and rectal cancer diagnosis. For colon cancer patients, median earnings drop from € 31,319 (95% confidence interval (CI): 30,161–32,478) in the year prior to diagnosis to € 23,924 (95% CI: 22,532–25,315) in the year after diagnosis. For rectal cancer patients, the corresponding drop is similar from € 32,636 (95% CI: 31,452–33,820) to € 22,647 (95% CI: 21,333–23,961). For disposable income, the decrease was much less pronounced.

The lower panel of Fig. 2 shows the difference in median annual earnings and disposable income between cancer patients and their comparators. In the year prior to diagnosis, there was practically no difference between colon cancer patients and their matched comparators, but rectal cancer patients had a marginally increased median income compared to their comparators, with a median difference in earnings of € 812 (95% CI: 304–1320) and a median difference in disposable income of € 349 (95% CI: 97–601). One year after diagnosis, both colon and rectal cancer patients had significantly lower earnings and disposable income than their matched comparators, the difference being much less pronounced for disposable income. For colon cancer, the difference was € −7989 (95% CI: −8999 to −6979) in earnings and € −1308 (95% CI: −1718 to −898) in disposable income. For rectal cancer, the difference was € −9463 (95% CI: −10,183 to −8743) in earnings and € −588 (95% CI: −858 to −319) in disposable income. For both disposable income and earnings, there was a statistically significant difference in the income drop between colon and rectal cancer patients in relation to their respective comparators, with p-values of 0.023 and < 0.0001 for disposable income and earnings, respectively. The difference in disposable income between colon cancer patients and their matched comparators was slightly more pronounced relative to rectal cancer patients. The difference in earnings between rectal cancer patients and their matched comparators was larger in the year after diagnosis compared with colon cancer patients. Sensitivity analyses restricted to individuals alive at least 3 years after diagnosis gave similar results (see Supplementary Figure S1). An interaction between sex, cancer exposure, and years from diagnosis/matching, was tested but did not yield statistical significance (p = 0.17 and p = 0.69 for earnings and disposable income respectively).

**Fig. 3:**
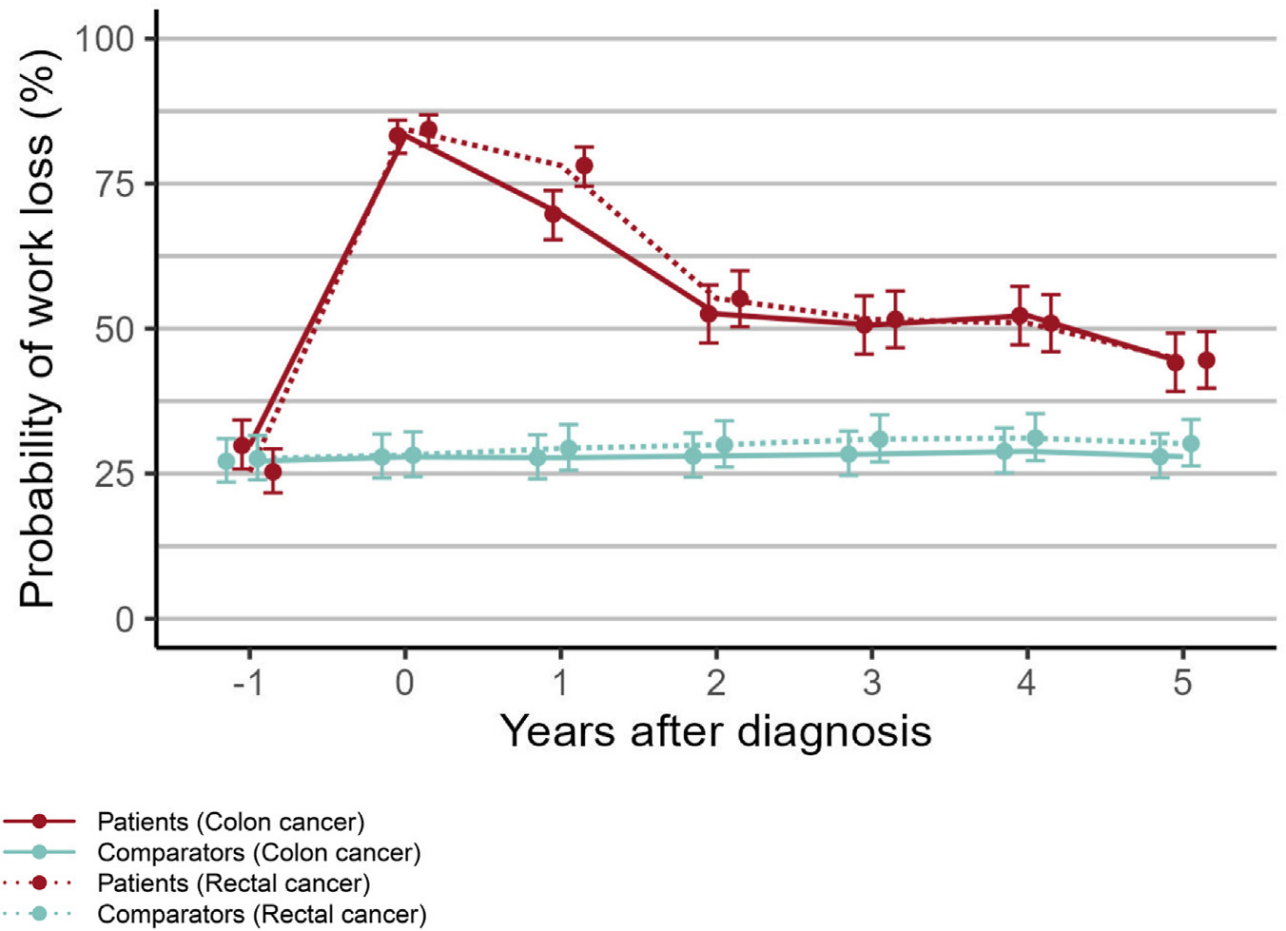
Yearly probability of work loss, starting the calendar year prior to diagnosis and ending in the fifth year after diagnosis, with year 0 being the calendar year of diagnosis. The probabilities are adjusted for age, sex, calendar date, and education level, with reference levels being age 52.5 years, calendar date 1st of July 2020, and upper secondary school education. Vertical lines show 95% confidence intervals.

The adjusted annual probability of work loss is shown in Fig. 3. For colon cancer and rectal cancer patients, the adjusted probability of any work loss in the year prior to diagnosis was 29.8% (95% CI: 25.8–34.2%) and 25.3% (95% CI: 21.7–29.3%), respectively, which was similar to their respective comparators. The year of diagnosis, colon and rectal cancer patients’ probabilities increased to 83.3% (95% CI: 80.2–86.0%) and 84.4% (95% CI: 81.5–86.9%), respectively, while they remained almost constant for the comparators. The probability of any work loss for colon cancer and rectal cancer patients, starting from the year of diagnosis, remained greater than for the comparators during the entire follow-up. When comparing the difference in probability of work loss between patients and comparators for colon cancer vs rectal cancer, a statistically significant difference (p < 0.0001) was found. This difference was most pronounced in the year after diagnosis with rectal cancer patients having a probability of 78.1% (95% CI: 74.6–81.3%) while colon cancer patients had a probability of 69.7% (95% CI: 65.3–73.8%), their respective comparators having around 28–29%. After five years, colon cancer patients had a probability of 44.1% (95% CI: 39.2–49.2%) while their comparators had a probability of 27.9% (95% CI: 24.3–31.9%), and rectal cancer patients had a probability of 44.6% (95% CI: 39.7–49.5%) while their comparators had a probability of 30.2% (95% CI: 26.3–34.3%). Sick leave and disability pension are shown as separate outcomes in Supplementary Figure S2, where the difference between patients and matched comparators was more pronounced for sick leave than for disability pension.

The mean number of months (unadjusted) of work loss each year of follow-up is displayed in Table 2, and separately for sick leave or disability pension in Supplementary Table S1. It is stratified into three groups: colon cancer, and rectal cancer with and without neoadjuvant radiotherapy. On average, colon cancer patients had 1.63 months of work loss in the year prior to diagnosis which increased to 3.67 months and 3.74 months in the year of diagnosis and the year after diagnosis, respectively. The corresponding figures for rectal cancer patients with and without neoadjuvant radiotherapy were 1.41, 4.37, 5.21 and 1.61, 3.61, 3.87, respectively. The greater mean months of work loss among rectal cancer patients who received neoadjuvant radiotherapy remained for the entire follow-up time.

**Table 2:** The mean number of months of work loss stratified into colon cancer patients, rectal cancer patients who did not undergo neoadjuvant radiotherapy, and rectal cancer patients who did undergo neoadjuvant radiotherapy.

Years since diagnosis	Colon cancer	Rectal cancer w/o neoadjuvant radiotherapy	Rectal cancer w neoadjuvant radiotherapy	Comparators
−1	1.63	1.61	1.41	1.59
0	3.67	3.61	4.37	1.67
1	3.74	3.87	5.21	1.72
2	2.65	2.96	3.58	1.77
3	2.60	2.97	3.40	1.83

## Discussion

In this nationwide cohort study of stage I-III colon and rectal cancer patients of working age, annual median earnings dropped after a CRC diagnosis, and did not recover fully during follow-up relative to comparators. However, there were only small differences in annual median disposable income between colon and rectal cancer patients and their respective comparators. The substantial drop in earnings as well as the substantial increase in work loss indicate the severity with which a CRC diagnosis and its treatment impairs the working abilities of patients. In addition to the impact on the individual with CRC, this also reflects a societal impact of CRC. On the other hand, disposable income directly measures the actual income loss for the patient. The observation that the patient cohort did not experience a severe loss of disposable income suggests that the loss of earnings was mitigated by benefits received through the Swedish welfare system.

The hypothesis that rectal cancer patients would suffer worse outcomes than colon cancer patients could not be confirmed with clarity. Changes in both disposable income and earnings did not differ substantially between rectal and colon cancer, by themselves or in relation to the respective comparators. However, the probability of work loss for CRC cases relative to comparators was higher for rectal cancer. There was no substantial difference in work loss between colon and rectal cancer patients without preoperative treatment, but rectal cancer patients that received neoadjuvant treatment experienced a greater work loss of about one month/year and the difference remained during follow-up. These results indicate that, at least for a sub-group, work loss may be a greater problem for rectal cancer patients. Cancer rehabilitation may decrease the amount of work loss, but given our results, work loss is likely to occur for several years postoperatively.

Indications for neoadjuvant therapy in rectal cancer are similar across Europe,^22^ but the treatment has been used more frequently in Sweden compared with the other Scandinavian countries.^23^ In addition, rectal cancer trials such as Stockholm III (NCT00904813) and RAPIDO (NCT01558921) included patients across Sweden between 1998 and 2016. During the studied period, neoadjuvant treatment was given to the most advanced tumors, i.e., higher T- and/or N-stage, and more distal tumors. Patients with these tumors are at a higher risk of more extensive surgery, permanent colostomy, as well as recurrences.

In addition, neoadjuvant therapy is associated with an increased risk of both early and late post-operative side-effects such as impaired bowel and urinary function.^24^ Thus, among rectal cancer patients having received neoadjuvant therapy and later a sphincter-preserving procedure, the increased incidence of post-therapeutic urogenital and bowel dysfunction may contribute to an increased risk of work loss. Furthermore, patients with a permanent stoma may need more health care and experience more work loss.^25^ Data from SCRCR show that 64% of rectal cancer patients having received pre-operative radiotherapy received a permanent stoma (abdominoperineal resection/Hartmann's resection), whereas anterior resection was more common in non-irradiated patients (70%).^26^ In our study, patients operated with abdominoperineal resection or Hartmann's resection collectively constituted about 1/3 of the rectal cancer cohort.

For colon cancer patients, in whom neoadjuvant therapy is uncommon, postoperative adjuvant chemotherapy is commonly prescribed to stage III or high-risk stage II patients. Currently, oxaliplatin containing chemotherapy regimens are often used and this may cause, sometimes permanent, disabling peripheral neuropathy in hands and feet.^27^ Oxaliplatin was approved in Europe already in 1996 but the first reports on its use in the adjuvant setting appeared almost 10 years later.^28^ In our dataset, colon cancer patients from 2007 and onwards were included and it is likely that oxaliplatin containing regimens were not fully implemented across Sweden during the earlier years of this study. It is possible that this has contributed to lower degree of work loss observed in our study.

Following a systematic literature review on financial toxicity including employment status in connection with various types of cancer, the authors found that cancer negatively affects employment.^29^ Only one study included in the review was specifically about CRC and that study analyzed 137 CRC survivors with a matched group from the general population of 355 individuals.^30^ Our study of almost 9000 patients and 53,000 comparators corroborates the findings that CRC is associated with work loss. Our study is set in Sweden where the welfare system almost fully compensates the reduction in earnings after a CRC diagnosis.

Strengths of this study include its nationwide coverage including Swedish rectal cancer patients from 1995 and Swedish colon cancer patients from 2007. Given the high-quality information on tumor characteristics in SCRCR, we were able to confidently identify patients with stage I-III disease. The LISA register has excellent coverage, with few missing data, earnings data based on reports to the Swedish Tax Authorities, and comprehensive data for the construction of disposable income. These measures allowed us to analyse trajectories for earnings and disposable income, as well as the extent to which patients were able to return to work after being cured. Comparing disposable income and earnings further allowed for assessment of the efficacy of Sweden's welfare system in its ability mitigate loss of earnings. A sensitivity analysis showed that this was not related to increased mortality among the “most vulnerable” — the successful mitigation of loss of earnings due to benefits was similar in a cohort where inclusion required surviving beyond the third year after diagnosis. In addition, there was limited loss to follow-up. CRC patients being conditionally similar to their matched comparators in the year preceding diagnosis for all outcomes suggests that the differences between patients and comparators from the year of diagnosis and onwards cannot be explained by confounding factors making the patients more susceptible to both income loss or work loss and CRC. Though welfare systems vary between countries, the results on loss of earnings and work loss can be cautiously generalized outside of Sweden, with the qualification that disposable income can possibly be more affected in countries outside of Sweden.

Limitations of this study include lack of data on treatment of patients after recurrence. Patients in whom a recurrence is detected may be subjected to additional surgery or palliative chemotherapy that can impact their ability to work. In a population-based report from Denmark on CRC patients stage I-III, rectal cancer patients had a higher recurrence risk compared to colon cancer patients.^31^ Thus, it is possible that among our cohorts there may be more rectal cancer patients who were not disease-free although they were survivors, and therefore became unable to work. As we censor observations at the time of a detected recurrence, we do not account for financial toxicity that may arise due to additional treatment after a recurrence.

Another limitation is that we do not measure all aspects of financial toxicity such as subjective financial distress and additional costs caused by the CRC. While disposable income remained largely unaltered after diagnosis, we are unable to make statements about the subjective experiences of these patients.

This study shows that despite work loss and decreased earnings, Swedish CRC patients have maintained disposable income from the year of diagnosis and 5 years onward. All CRC patients experience an increased probability of work loss following diagnosis, especially during the first 2 years, and especially rectal cancer patients who received neoadjuvant therapy. Post-therapeutic support should include actions to help patients continue to work.

## Contributors

All authors contributed to the study conception and design. Data collection for CRCBaSe was performed by Caroline Nordenvall, Sol Erika Boman, and Ida Hed Myrberg. Data for CRCBaSe was verified by Caroline Nordenvall, Sol Erika Boman and Ida Hed Myrberg. The data for this manuscript was accessed by Sol Erika Boman and Ida Hed Myrberg. The first draft of the manuscript was written by Sol Erika Boman, Ida Hed Myrberg, Per J Nilsson, Anna Martling, Gustaf Bruze and Caroline Nordenvall. All authors commented on all previous versions of the manuscript. All authors read and approved the final manuscript.

## Data sharing statement

Individual-level data used for the analysis in this study cannot be shared with others due to EU's GDPR and Swedish research legislation.

## Declaration of interests

All authors declare no competing interests.

## Appendix A Supplementary data

Supplementary Figure S1 Median annual income (top panel) and difference in median annual income between CRC cases and matched controls (bottom panel), starting the calendar year prior to diagnosis and ending after the third year after diagnosis, with year 0 being the calendar year of diagnosis, converted to euros and inflation-adjusted to represent the value of a euro in the year 2020. The levels and differences are adjusted for age, sex, calendar date, and education level, with reference levels being age 52.5 years, calendar date 1st of July 2020, and upper secondary school education. Vertical lines show 95% confidence intervals. Sensitivity analysis where patients and their matched comparators were required to be alive for at least three years after the year of diagnosis.

Supplementary Figure S2 Yearly probability of being on sick leave (the upper panel) or of being on disability pension (the lower panel), starting the calendar year prior to diagnosis and ending after the fifth year after diagnosis, with year 0 being the calendar year of diagnosis. The probabilities are adjusted for age, sex, calendar date, and education level, with reference levels being age 52.5 years, calendar date 1st of July 2020, and upper secondary school education. Vertical lines show 95% confidence intervals.
