# Supplementary figures and images for: Earnings and work loss after colon and rectal cancer: a Swedish nationwide matched cohort study
Source: eClinicalMedicine. 2024 Aug 6;75:102770. doi: 10.1016/j.eclinm.2024.102770 (PMC11359760; doi:10.1016/j.eclinm.2024.102770)

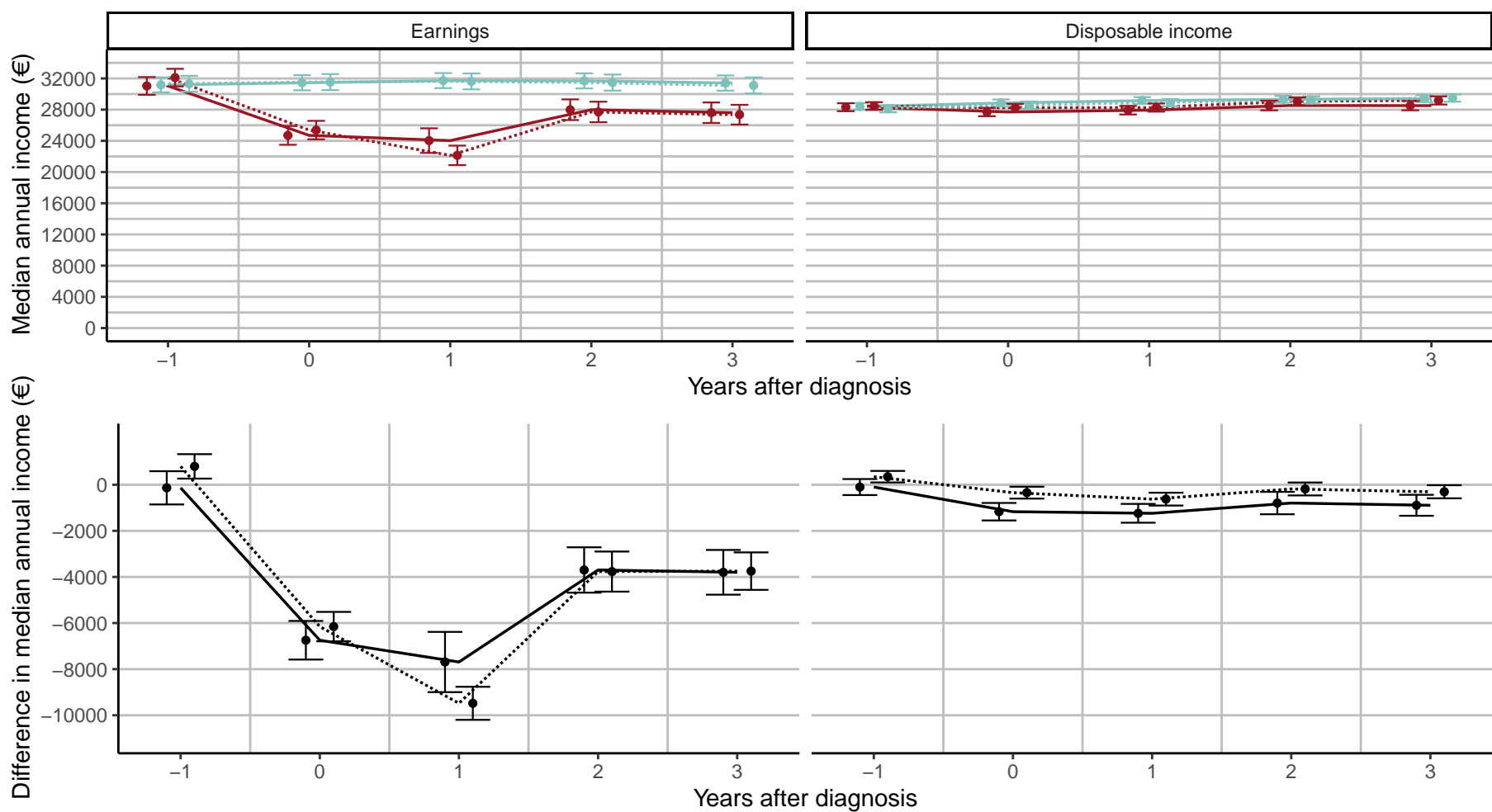

Supplement: Supplementary Figure S1 — Median annual income (top panel) and difference in median annual income between CRC cases and matched controls (bottom panel), starting the calendar year prior to diagnosis and ending after the third year after diagnosis, with year 0 being the calendar year of diagnosis, converted to euros and inflation-adjusted to represent the value of a euro in the year 2020. The levels and differences are adjusted for age, sex, calendar date, and education level, with reference levels being age 52.5 years, calendar date 1st of July 2020, and upper secondary school education. Vertical lines show 95% confidence intervals. Sensitivity analysis where patients and their matched comparators were required to be alive for at least three years after the year of diagnosis. [file mmc1.pdf]

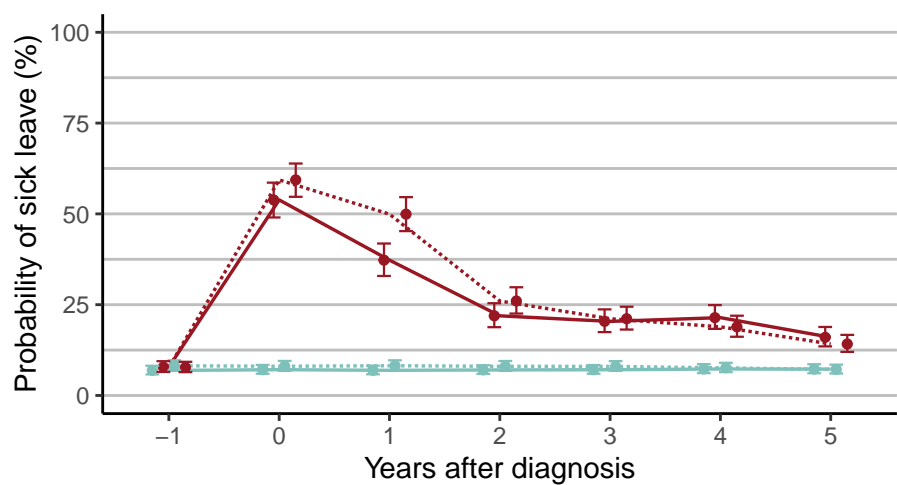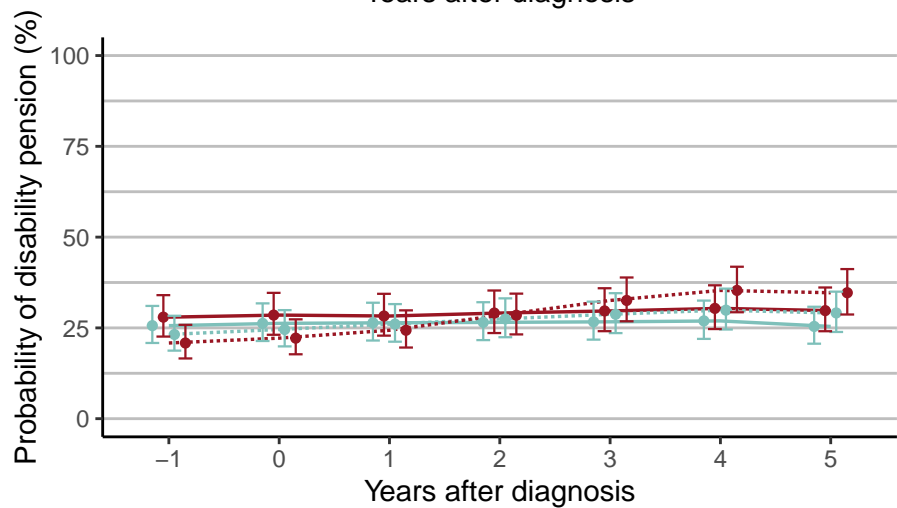

Supplement: Supplementary Figure S2 — Yearly probability of being on sick leave (the upper panel) or of being on disability pension (the lower panel), starting the calendar year prior to diagnosis and ending after the fifth year after diagnosis, with year 0 being the calendar year of diagnosis. The probabilities are adjusted for age, sex, calendar date, and education level, with reference levels being age 52.5 years, calendar date 1st of July 2020, and upper secondary school education. Vertical lines show 95% confidence intervals. [file mmc2.pdf]
