# Supplementary material for: Earnings and work loss after colon and rectal cancer: a Swedish nationwide matched cohort study
Source: eClinicalMedicine. 2024 Aug 6;75:102770. doi: 10.1016/j.eclinm.2024.102770 (PMC11359760; doi:10.1016/j.eclinm.2024.102770)
Supplement: Supplementary Table [file mmc3.docx]

|  | **Sick leave (months)** | | | | **Disability leave (months)** | | | |
| --- | --- | --- | --- | --- | --- | --- | --- | --- |
| **Years since diagnosis** | **Colon cancer** | **Rectal cancer w/o neoadjuvant radiotherapy** | **Rectal cancer w neoadjuvant radiotherapy** | **Comparators** | **Colon cancer** | **Rectal cancer w/o neoadjuvant radiotherapy** | **Rectal cancer w neoadjuvant radiotherapy** | **Comparators** |
| -1 | 0.41 | 0.48 | 0.40 | 0.43 | 1.22 | 1.12 | 1.01 | 1.16 |
| 0 | 2.43 | 2.38 | 3.28 | 0.44 | 1.24 | 1.23 | 1.09 | 1.22 |
| 1 | 2.48 | 2.54 | 4.01 | 0.44 | 1.26 | 1.34 | 1.20 | 1.28 |
| 2 | 1.38 | 1.42 | 2.17 | 0.43 | 1.27 | 1.54 | 1.41 | 1.34 |
| 3 | 1.30 | 1.22 | 1.71 | 0.44 | 1.30 | 1.75 | 1.69 | 1.39 |
| 4 | 1.34 | 1.12 | 1.55 | 0.43 | 1.34 | 1.87 | 1.86 | 1.43 |
| 5 | 1.12 | 0.89 | 1.19 | 0.41 | 1.31 | 1.78 | 1.80 | 1.37 |

**Supplementary Table 1: The mean number of months on sick leave and disability pension, respectively, stratified into colon cancer patients, rectal cancer patients who did not undergo neoadjuvant radiotherapy, and rectal cancer patients who did undergo neoadjuvant radiotherapy.**
